# Long‐term outcome following multimodality treatment in a cat with recurrent laryngeal adenocarcinoma

**DOI:** 10.1111/vru.13466

**Published:** 2024-12-16

**Authors:** Christine Sesanto, Jessica Lawrence, Jocelyn Holkham, Juan Carlos Serra, Spela Bavcar, Magdalena Parys

**Affiliations:** ^1^ Cancer Care Centre Willows Veterinary Centre & Referral Service Solihull UK; ^2^ Department of Surgical and Radiological Sciences School of Veterinary Medicine University of California Davis California USA; ^3^ The Hospital for Small Animals The University of Edinburgh Royal Dick School of Veterinary Studies Edinburgh UK; ^4^ Small Animal Specialist Hospital Cancer Centre Sydney Australia

**Keywords:** carcinoma, feline, larynx, polyp, radiotherapy

## Abstract

A 9.5‐year‐old male neutered domestic short‐haired cat received two courses of postoperative, definitive‐intent conformal radiation therapy (RT) for recurrent laryngeal adenocarcinoma (LACA). Adjuvant RT was prescribed (16 × 3.0 Gy, total 48 Gy) following incomplete resection. Following tumor recurrence and subsequent incomplete resection 31.5 months after the first course, a second course was prescribed (20 × 2.5 Gy, total 50 Gy). Acute and late adverse events were mild. No evidence of local recurrence was documented 42 months following the second course when the cat was euthanized for renal disease. This first report of long‐term control in a cat with LACA supports further evaluation of surgery and definitive‐intent RT.

## 1 SIGNALMENT, HISTORY, AND CLINICAL FINDINGS

A 9.5‐year‐old male neutered DSH cat (4.18 kg) was referred to the Royal (Dick) School of Veterinary Studies R(D)SVS Radiation Oncology service for adjunctive definitive‐intent radiation therapy (RT) for a well‐differentiated laryngeal adenocarcinoma (LACA). A friable, ulcerated, soft‐tissue mass arising from the ventral larynx was identified on oral examination by the referring veterinarian (rDVM) following 3 months duration of progressive upper respiratory tract (URT) noise. Three debulking surgeries were subsequently performed 16, 8.5, and 1.5 months prior to referral, with temporally associated histopathologic progression consisting of laryngeal polyp, adenoma, and well‐differentiated adenocarcinoma, respectively. Because the second and third resections were performed to alleviate acute and severe tumor‐associated airway obstruction, and histopathology confirmed a well‐differentiated adenocarcinoma, the cat was referred for postoperative RT.

Initial physical examination, complete blood count, biochemistry, and cytology of mandibular lymph nodes on both sides at R(D)SVS revealed no clinically relevant abnormalities. Pre‐ and postcontrast CT images of the head, neck, and thorax were acquired using a four‐slice helical CT (Volume Zoom, Siemens) using 2 mm axial slice thickness. Following the acquisition of precontrast CT, intravenous (IV) contrast was administered (2 mL/kg Omnipaque 350; GE Healthcare, Chalfont St Giles), and postcontrast images were acquired. CT did not identify lymphadenomegaly or lymphadenopathy, pulmonary metastasis, or other abnormalities.

## 2 TREATMENT AND OUTCOME

During CT image acquisition, the cat was positioned in left‐lateral recumbency, head facing the gantry, with a vacuum‐formable mattress (Vac‐Loc; Civco Medical Solutions) for immobilization and reproducible positioning. A 3 mm tissue‐equivalent bolus was applied over the anticipated irradiation field (i.e., ventrolateral neck). Gross tumor volume (GTV) was not defined as the larynx was normal on CT. The clinical target volume (CTV) encompassed a 3 mm isotropic expansion around the laryngeal soft tissues, including the prior surgical site, which was visible on the oral exam. Due to the lateral positioning, an additional expansion (3 mm cranial, 9 mm caudal) was added to create the planning target volume (PTV). Organs at risk included the brain (cerebrum, cerebellum, brainstem), bullae, esophagus, eyes, spinal cord, and trachea (Table 1). Within the radiation planning system (Varian Eclipse v11; Varian Medical Systems), 48 Gy in 16 fractions was prescribed to isocenter within the PTV by a board‐certified radiation oncologist (J.L.). Plan acceptance required that 95% of the PTV was covered by 95% of the prescription dose with the maximum dose less than 115% of the dose. Two opposing conformal (120‐leaf multileaf collimator) 6MV fields were sufficient for plan goals. Radiation was delivered using a Varian linear accelerator (Clinac C/D 600, Varian Medical Systems), and positioning was verified prior to each treatment with the use of an onboard MV‐electronic portal imaging device.

**TABLE 1:** Volume in centimeters cubed (cm^3^) of target volumes and organs at risk used in the calculation and assessment of the first (C1) and second (C2) radiotherapy courses.

RT course	Volume of targets (cm^3^)		Volume of organs at risk (cm^3^)						
	CTV	PTV	Brain^a^	Bulla left	Bulla right	Eso	OD	OS	Tra
C1	0.95	3.76	23.66	0.94	0.91	0.71	4.53	5.03	3.49
C2	6.26	18.86	21.39	0.84	1.01	0.70	5.65	5.23	3.13

Abbreviations: CTV, clinical target volume; Eso, esophagus; OD, right eye; OS, left eye; PTV, planning target volume; Tra, trachea.

^a^ Brain (included: cerebrum, cerebellum, and brain stem).

RT commenced 1 week following the CT simulation. Prednisolone (0.5 mg/kg PO *q*48 h) was prescribed to reduce the risk of intubation or treatment‐related inflammation. Radiation fractions were administered on an outpatient basis, once daily on weekdays, except for fractions 8 and 9 (given 6 h apart for scheduling needs). An IV cannula was placed, maintained, and removed once weekly to reduce patient stress and time in the hospital. Mild laryngeal swelling and focal petechiae were incidentally noted during intubation at fraction 9; this resolved after switching to a smaller endotracheal tube and increasing prednisolone administration frequency (*q*24 h). Gabapentin (12 mg/kg PO *q*12 h) was added after fraction 11 for analgesia in anticipation of radiation‐induced laryngitis and mucositis. Grade 1 acute radiation toxicity of the skin and oral mucous membranes developed after fraction 13 and resolved two weeks following RT completion. Gabapentin was discontinued upon resolution of acute effects, and prednisolone was tapered over 60 days. Grade 1 cutaneous late RT toxicity was characterized by alopecia, leukotrichia, and mild hyperpigmentation.^1^

The owner reported excellent quality of life (QoL) during and after RT. However, thirty months after starting her first RT treatment, the owner noted increased URT noise during sleep. Upon oral exam with the rDVM, an ulcerated, friable laryngeal mass at the previous excision site was identified and marginally removed. Histopathology performed by a board‐certified pathologist confirmed a laryngeal carcinoma. Repeat CT of the head, neck, and thorax did not reveal metastatic disease. Complete blood count, biochemistry, and urinalysis revealed renal insufficiency (IRIS stage 2) and mild anemia (HCT 25.7, RI 27–50).

The cat presented to the radiation oncology service 1 month postoperatively for a CT for RT planning and diagnostic abdominal CT to rule out metastasis. Pre‐ and postcontrast images were acquired with a 64‐slice helical CT scanner (Definition AS, Siemens). The cat was positioned in left lateral recumbency, immobilized with a vacuum‐formable mattress (Vac‐Loc; Civco Medical Solutions), and a 5 mm tissue‐equivalent bolus was applied to the ventrolateral neck. CT evaluation was normal, with the exception of a 5 mm diameter contrast‐enhancing soft tissue thickening of the ventral larynx that partially narrowed the airway lumen. A board‐certified soft‐tissue surgeon performed same‐day cytoreductive surgery 30 days after the previous rDVM resection. The lesion was friable with a cauliflower‐like appearance, and there were no visible signs of laryngeal late adverse events in the larynx. Histopathology performed by a different board‐certified pathologist was consistent with recurrent, poorly differentiated LACA. CT for radiation planning was repeated ten days postsurgery in the same left lateral position with a 5 mm tissue‐equivalent bolus. The CT revealed a 3.5 mm diameter contrast‐enhancing thickening at the ventral larynx, most consistent with postoperative inflammation, which was not visible on the laryngeal exam; GTV was, therefore, not defined. The CTV encompassed the laryngeal soft tissues to include the contrast‐enhanced thickening at the surgical site and expanded craniocaudally within the soft tissue to create a 45 mm long target volume. A 3 mm isotropic expansion around the CTV was used to generate the PTV. The same organs at risk were contoured as with the initial course. Radiation planning was performed by a board‐certified radiation oncologist (M.P.); 50 Gy in 20 fractions was prescribed to the isocenter within PTV. An approved plan was created with the use of two parallel‐opposed, conformal fields, ensuring 95% of the PTV was covered by 95% of the prescription dose. Daily pretreatment MV portal imaging was done to verify proper positioning. Fractions were administered once daily, Monday through Friday, on an in‐patient basis for owners’ convenience. Medications prescribed included prednisolone (0.5 mg/kg *q*24 h) at the start of RT, mirtazapine (0.5 mg/kg *q*48 h as needed), gabapentin (12 mg/kg PO *q*12 h) after fraction 10, and transmucosal buprenorphine (20mcg/kg *q*8‐12 h) after fraction 11. Grade 1 acute and late radiation toxicity of the skin was present at the time of the last RT fraction and characterized by mild erythema, alopecia, leukotrichia, and mild hyperpigmentation.^1^ All medications were tapered within 3 weeks following the last RT fraction.

Following discharge from the second RT course, primary monitoring of the cat's response was managed with intermittent rDVM examinations and client email updates to the RT service; excellent quality of life was reported. The cat was euthanized due to end‐stage renal disease and hypertension 75.5 months after the initial diagnosis of well‐differentiated LACA; this equates to 74 months after starting the first RT course and 42 months after starting the second course. Restaging by the rDVM immediately prior to euthanasia, including a laryngeal exam, lymph node palpation, thoracic radiographs, and abdominal ultrasound, showed no evidence of LACA recurrence or spread; the previously noted grade 1 late RT toxicities remained static.

## 3 DISCUSSION

Laryngeal tumors are rare in cats, with lymphoma being the most common, followed by squamous cell carcinoma (SCC).^2^, ^3^, ^4^, ^5^, ^6^, ^7^, ^8^ Local or distant metastasis is reportedly rare in malignant tumors.^7^, ^9^ Benign laryngeal masses also occur in cats, including cysts, lymphoplasmacytic inflammation, polypoid laryngitis, and lymphoid hyperplasia.^2^, ^3^, ^4^, ^7^, ^8^, ^10^, ^11^, ^12^ Clinical signs are usually consistent with upper airway obstruction but can also include dysphonia, dysphagia, weight loss, anorexia, exercise intolerance, oral hemorrhage, and ptyalism.^3^, ^4^, ^6^, ^7^, ^8^

There is sparse veterinary literature to guide optimal treatment paradigms. The prognosis is considered guarded, with most feline patients being euthanized at the time of diagnosis or days after intervention due to rapid clinical deterioration.^2^, ^4^, ^7^, ^9^ While small laryngeal lesions may be removed by mucosal resection or partial laryngectomy.^2^ surgery alone for malignant laryngeal tumors is often palliative.^2^, ^3^, ^7^ In a study describing partial laryngectomy and tracheostomy tube placement, one cat with laryngeal carcinoma was euthanized seven days postoperatively due to severe respiratory distress caused by recurring tracheostomy tube obstruction.^2^ Total laryngectomy with permanent tracheostomy for a large laryngeal peripheral nerve sheath tumor was described in one cat, with postoperative survival greater than 13 months.^13^ In a separate feline study, a total laryngectomy with permanent tracheostomy was performed for the treatment of upper airway obstruction.^12^ In a cohort of 21 cats, three were diagnosed with SCC, and one cat had a laryngeal mass of unknown etiology, although outcomes were not reported.^12^ In another study, 4 out of 23 cats with laryngeal SCC underwent permanent tracheostomy to improve respiratory compromise; two of these cats received RT of unknown fractionation.^14^ Euthanasia was performed for 281 days in one cat due to anorexia, and another cat died of occlusion of the tracheostomy site 2 days after permanent tube placement.^14^ The two cats that did not receive RT died from airway occlusion and were euthanized for anorexia 42 days and 7 days postprocedure, respectively.^14^ In a study of 27 cats, which included four cats with laryngeal SCC and three cats with tracheal masses, tracheostomy tube placement following diagnosis yielded survival times ranging from 1–6 days, with a median of 3 days; all cats died due to rapid disease progression.^4^ Collectively, these cases represent advanced disease, given the need for urgent tracheostomy tube placement and poor long‐term control.^12^, ^14^, ^15^

The role of chemotherapy, targeted drug therapies, and nonsteroidal anti‐inflammatory drugs (NSAIDs) has not been defined for feline LACA. Nonsteroidal anti‐inflammatory drugs, which reduce COX‐expression, are often used to manage various carcinoma types that overexpress COX‐2 and can be considered for LACA.^16^, ^17^ Possible adverse effects, such as renal toxicity, should be weighed against the significant benefits of NSAIDs in these patients; the risk of renal toxicity can be mitigated, with regular monitoring of renal parameters, appropriate patient selection, and use of established dosing regimens.^18^, ^19^ Out of four cats that received multimodality treatment, the one cat with LACA underwent tube tracheostomy, laryngectomy, and chemotherapy with doxorubicin and methotrexate; while this cat's specific survival time was not reported, the MST of the four cats was 134.5 days (range 80–183 days).^4^ Another cat with LACA was euthanized 2 months after partial resection and prednisolone.^8^ Prednisolone may improve respiratory function, as two cats with laryngeal SCC were euthanized 40 and 52 days after diagnosis and institution of prednisolone therapy.^3^

While the longest survival times in human LACA are achieved with combined surgery and RT.^20^ the efficacy of postoperative RT for feline LACA is unknown. There is a strong rationale to consider multimodality locoregional therapy, given the importance of local control for LACA. Based on the limited data available, long‐term survival with good QoL can be achieved with the use of surgery, radiation therapy, or a combination thereof in cats with laryngeal tumors.^3^, ^11^, ^14^ The cat described here was unique from prior literature in that routine monitoring and local treatment were provided from the time of diagnosis of laryngeal adenoma. From the time of disease progression to histopathologically confirmed well‐differentiated LACA, the cat achieved tumor control for 30 months after his first RT fraction. Early local progression and histopathologic confirmation of poorly differentiated LACA at that time led to repeat intervention with surgical resection and RT, providing more than 42 months of additional tumor control after starting the second RT course. Histopathology examinations were performed by multiple board‐certified veterinary pathologists at two different laboratories. While the specific prevalence and underlying mechanisms of tumorigenesis from adenoma to LACA are unknown, possible contributing factors may include inflammation, recurrence, and selection for acquired mutations favoring progression and therapeutic resistance and disrupted tumor microenvironment.^18^, ^21^

Two courses consisting of conventionally fractionated RT were well‐tolerated in this cat with LACA. To the authors’ knowledge, repeated definitive‐intent RT to the laryngeal region following 2 years of initial tumor control has not been previously described. Re‐irradiation with hypofractionated stereotactic or palliative protocol has been described in cats with nasal tumors and pituitary macroadenoma.^22^, ^23^, ^24^ Primary concerns with repeated RT include decreased duration of response compared with the initial RT course and injury to normal tissues surrounding the radiation target region. There is limited information regarding early and late toxicities following reirradiation in the feline head and neck.^22^, ^23^ Factors that are important in the prediction of permanent, irreversible late toxicities include radiation dose per fraction, total dose per course, lifetime dose to organs at risk, interval between courses, and repair capability of normal tissues. For acutely responding tissues, such as oral mucosa and skin, repair of radiation injury is more likely with a lower dose per fraction and prolonged interval between courses.^25^ Late‐responding tissues such as cartilage, bone, or nerves have less recovery capability compared with acutely responding tissues, thus raising concern for late radiation‐induced changes such as tissue necrosis.^25^

This cat was treated with 3D‐conformal radiation therapy (CRT), which provides a relatively conformal radiation plan with a prescription dose focused on the PTV. Megavoltage orthogonal portal images were used to verify the correct patient position prior to treatment. Because of the parallel opposed, conformal nature, the musculature and cartilages of the larynx, as well as the proximal esophagus and distal tongue, received moderate doses as the laryngeal epithelium at risk for tumor recurrence was targeted. This cat was prescribed a total dose of 98 Gy, with a 31.5‐month interval between the two courses, to the laryngeal tumor bed using fraction sizes of 2.5–3.0 Gy (Table 2). The cat was monitored for the most likely clinically significant radiation‐induced late toxicities, including lingual fibrosis, laryngeal dysfunction, osteoradionecrosis, chondroradionecrosis, chronic esophagitis, and esophageal stricture. This cat did not develop laryngeal or esophageal dysfunction despite two conventionally fractionated radiation courses. At the time of euthanasia, only cosmetic, grade 1 skin toxicity characterized by alopecia, leukotrichia, and mild hyperpigmentation were documented. However, without postmortem histopathology, proximal esophageal and laryngeal radiation injury cannot be fully excluded.

**TABLE 2:** Radiation dose statistics for radiation target volumes and organs at risk in centigray (cGy) from the first (C1) and second (C2) course of radiation therapy.

RT course	Dose statistic tab	Dose to targets (cGy)		Dose to organs at risk (cGy)						
		CTV	PTV	Brain^a^	Bulla left	Bulla right	Eso	OD	OS	Tra
C1	Min	4658	4480	9	47	33	9	5	5	9
	Max	4802	4809	79	367	175	4473	14	17	4800
	Mean	4749	4722	20	117	83	1548	9	10	2071
	Median	4752	4727	17	100	77	209	9	11	570
C2	Min	4851	4568	24	334	226	998	15	19	44
	Max	5217	5217	2039	4275	4250	4979	37	46	5166
	Mean	5093	5014	93	2360	1923	4192	24	29	2956
	Median	5100	5022	53	2303	1795	4697	23	28	4134

Abbreviations: CTV, clinical target volume; Eso, esophagus; OD, right eye; OS, left eye; PTV, planning target volume; Tra, trachea.

^a^ Brain (included: cerebrum, cerebellum, and brain stem).

Sesanto, Lawrence, Holkham, Serra, Bavcar, Parys

Advances in radiation planning techniques like intensity‐modulated RT or volumetric‐modulated arc therapy generate highly conformal plans, decreasing the risk of clinically significant RT toxicities. Image‐guided treatments to confirm target positioning prior to treatment further decrease the volume of normal tissue irradiated. Further investigation into advanced techniques that preserve laryngeal function is needed.

In conclusion, this is the first report of multimodality treatment achieving long‐term control of recurrent feline LACA. This is also the first reported feline LACA case to receive two courses of definitive‐intent 3D‐CRT following cytoreductive surgeries. No clinically relevant early or late toxicities were present after two courses of RT, and QoL was maintained for many years. Multimodal treatment may be considered after diagnosis of LACA to potentially obtain durable tumor control. Prospective studies on LACA in cats treated with multimodal therapy are needed to validate this approach.

## LIST OF AUTHOR CONTRIBUTIONS

### Category 1

(a) Conception and design: Sesanto, Parys, Lawrence
(b) Acquisition of data: Sesanto, Parys, Lawrence, Bavcar, Serra, Holkham
(c) Analysis and interpretation of data: Sesanto, Parys, Lawrence, Bavcar, Serra, Holkham

### Category 2

(a) Drafting the article: Sesanto, Parys, Lawrence, Bavcar, Serra, Holkham
(b) Reviewing article for intellectual content: Sesanto, Parys, Lawrence, Bavcar, Serra, Holkham

### Category 3

(a) Final approval of the completed article: Sesanto, Parys, Lawrence, Bavcar, Serra, Holkham

### Category 4

(a) Agreement: Sesanto, Parys, Lawrence, Bavcar, Serra, Holkham

## CONFLICT OF INTEREST STATEMENT

The authors declare no conflict of interest.

## DATA ACCESSIBILITY STATEMENT

The data used in the study are not available.

## ETHICS STATEMENT

This study complies with the Committee on Publication Ethics (COPE) guidelines and the RT protocols used in this case follow standard of care for veterinary patients. Animal owners provided written consent for the treatment provided and for the publication of data.

## PREVIOUS PRESENTATION OR PUBLICATION DISCLOSURE

ESVONC oral poster presentation (May 2021)

## REPORTING CHECKLIST DISCLOSURE

None was used.
